# 1-Phenyl-1*H*-naphtho­[1,2-*e*][1,3]oxazin-3(2*H*)-one

**DOI:** 10.1107/S1600536810035841

**Published:** 2010-09-11

**Authors:** Humaira Y. Gondal, Misbah Bhatti, Azra Gohar, Muhammad Ali, M. Nawaz Tahir

**Affiliations:** a Department of Chemistry, University of Sargodha, Sargodha, Pakistan; b Department of Physics, University of Sargodha, Sargodha, Pakistan

## Abstract

In the title compound, C_18_H_13_NO_2_, the naphthalene (r.m.s. deviation = 0.025 Å) and benzaldehyde (r.m.s. deviation = 0.006 Å) groups are oriented at a dihedral angle of 89.48 (4)°. The oxazine group is oriented at dihedral angles of 13.36 (4) and 85.08 (5)°, respectively, with respect to the naphthalene and benzaldehyde fragments. In the crystal, inversion dimers linked by pairs of C—H⋯O hydrogen bonds generate *R*_2_^2^(8) loops. The dimers are linked into [010] chains *via* N—H⋯O hydrogen bonds. Weak C—H⋯π links and aromatic π–π stacking between the centroids of the naphthalene phenyl rings [centroid–centroid separation = 3.5977 (8) Å] help to consolidate the packing.

## Related literature

For background to oxazinones, see: Patel *et al.* (1999 ▶); Waxman & Darke (2000 ▶). For graph-set notation, see: Bernstein *et al.* (1995 ▶). 
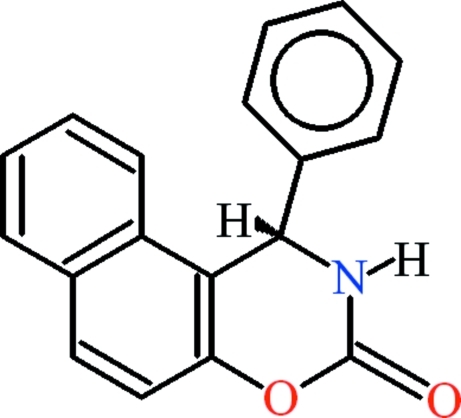


## Experimental

### 

#### Crystal data

C_18_H_13_NO_2_
*M* *_r_* = 275.29
Monoclinic, 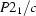

*a* = 11.5625 (4) Å
*b* = 16.9228 (5) Å
*c* = 7.2394 (2) Å
β = 98.155 (1)°
*V* = 1402.21 (7) Å^3^
*Z* = 4
Mo *K*α radiation
μ = 0.09 mm^−1^
*T* = 296 K
0.32 × 0.22 × 0.22 mm

#### Data collection

Bruker Kappa APEXII CCD diffractometer
Absorption correction: multi-scan (*SADABS*; Bruker, 2005 ▶) *T*_min_ = 0.980, *T*_max_ = 0.982
9236 measured reflections
2533 independent reflections
1961 reflections with *I* > 2σ(*I*)
*R*_int_ = 0.022

#### Refinement

*R*[*F* ^2^ > 2σ(*F* ^2^)] = 0.039
*wR*(*F* ^2^) = 0.111
*S* = 1.03
2533 reflections
190 parameters
H-atom parameters constrained
Δρ_max_ = 0.16 e Å^−3^
Δρ_min_ = −0.15 e Å^−3^

### 

Data collection: *APEX2* (Bruker, 2009 ▶); cell refinement: *SAINT* (Bruker, 2009 ▶); data reduction: *SAINT*; program(s) used to solve structure: *SHELXS97* (Sheldrick, 2008 ▶); program(s) used to refine structure: *SHELXL97* (Sheldrick, 2008 ▶); molecular graphics: *ORTEP-3 for Windows* (Farrugia, 1997 ▶) and *PLATON* (Spek, 2009 ▶); software used to prepare material for publication: *WinGX* (Farrugia, 1999 ▶) and *PLATON*.

## Supplementary Material

Crystal structure: contains datablocks global, I. DOI: 10.1107/S1600536810035841/hb5635sup1.cif

Structure factors: contains datablocks I. DOI: 10.1107/S1600536810035841/hb5635Isup2.hkl

Additional supplementary materials:  crystallographic information; 3D view; checkCIF report

## Figures and Tables

**Table 1:** Hydrogen-bond geometry (Å, °) *Cg*2 is the centroid of the C1–C6 phenyl ring.

*D*—H⋯*A*	*D*—H	H⋯*A*	*D*⋯*A*	*D*—H⋯*A*
N1—H1⋯O2^i^	0.86	2.07	2.8698 (17)	155
C8—H8⋯O1^ii^	0.93	2.58	3.4725 (18)	161
C16—H16⋯*Cg*2^iii^	0.93	2.92	3.722 (2)	145

Symmetry codes: (i) 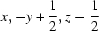; (ii) 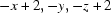; (iii) 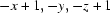.

**Fig. 1.**
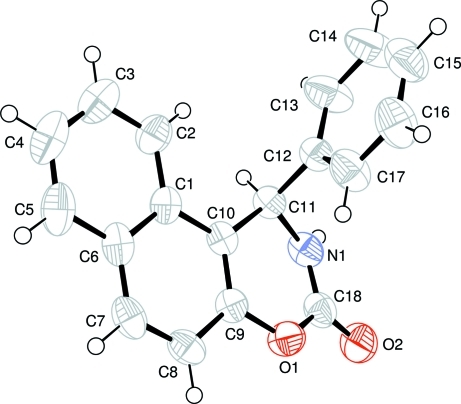
View of (I) with displacement ellipsoids drawn at the 50% probability level. H-atoms are shown as small spheres of arbitrary radii.

**Fig. 2.**
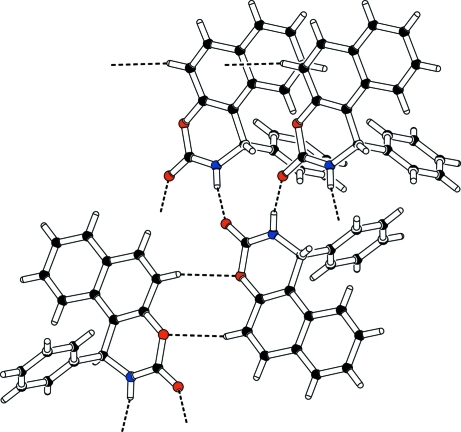
The partial packing of (I), which shows that molecules are dimerized which are interlinked in one dimensional infinite polymeric chains.

## supplementary crystallographic information

### Comment

Oxazinones are an important class of heterocyclic compounds with a diverse range of biological activities (Patel *et al.*, 1999; Waxman & Darke, 2000). During recent studies for the search of efficient, simple and green method for the preparation of naphthalene-condensed 1,3-oxazin-3-one derivatives, we have obtained the title compound (I, Fig. 1).

In the title compound, the naphthalene group A (C1—C10) and moiety B (C11—C17) of benzaldehyde group are planar with r. m. s. deviations of 0.0252 and 0.0056 Å, respectively. The dihedral angle between A/B is 89.48 (4)°. The fused group C (O1/C18/O2/N1) is also planar with r. m. s. deviation of 0.0037 Å. The dihedral angle between A/C and B/C is 13.36 (4) and 85.08 (5)°, respectively. The title compound is dimerized due to H-bonding of C—H···O type (Table 1, Fig. 2) with *R*_2_^2^(8) ring motifs (Bernstein *et al.*, 1995). The dimers are interlinked due to N—H···O type of H-bondings (Table 1, Fig. 2). There exists π–π interaction between the centroids of phenyl rings (C1/C6—C10) at a distance of 3.5977 (8) Å [symmetry code: 2 - *x*, - *y*, 1 - *z*]. The molecules are stabilized in the form of infinite one dimensional polymeric chains extending along the *c* axis.

### Experimental

A mixture of β-naphthol (1.0 mmol), benzaldehyde (1.0 mmol), urea (1.0 mmol) and CuCl_2_ (0.1 mm mol) as a catalyst, were heated at 393 K in a round bottom flask for 3 h. The reaction was monitored through TLC. After completion of the reaction, the mixture was cooled to room temperature and washed thoroughly with distilled water. The crude product obtained was recrystallized from petroleum ether:ethyl acetate (1:5) to affoard colourless rods of (I) after 24 h.

### Refinement

The H-atoms were positioned geometrically (C–H = 0.93–0.96 Å) and were included in the refinement in the riding model approximation, with *U*_iso_(H) = x*U*_eq_(C), where x = 1.5 for methyl and x = 1.2 for aryl H-atoms.

### Crystal data

**Table:**

C_18_H_13_NO_2_	*F*(000) = 576
*M**_r_* = 275.29	*D*_x_ = 1.304 Mg m^−^^3^
Monoclinic, *P*2_1_/*c*	Mo *K*α radiation, λ = 0.71073 Å
Hall symbol: -P 2ybc	Cell parameters from 1961 reflections
*a* = 11.5625 (4) Å	θ = 2.2–25.3°
*b* = 16.9228 (5) Å	µ = 0.09 mm^−^^1^
*c* = 7.2394 (2) Å	*T* = 296 K
β = 98.155 (1)°	Rod, colourless
*V* = 1402.21 (7) Å^3^	0.32 × 0.22 × 0.22 mm
*Z* = 4	

### Data collection

**Table:**

Bruker Kappa APEXII CCD diffractometer	2533 independent reflections
Radiation source: fine-focus sealed tube	1961 reflections with *I* > 2σ(*I*)
graphite	*R*_int_ = 0.022
Detector resolution: 8.10 pixels mm^-1^	θ_max_ = 25.3°, θ_min_ = 2.2°
ω scans	*h* = −13→13
Absorption correction: multi-scan (*SADABS*; Bruker, 2005)	*k* = −18→20
*T*_min_ = 0.980, *T*_max_ = 0.982	*l* = −8→6
9236 measured reflections	

### Refinement

**Table:**

Refinement on *F*^2^	Primary atom site location: structure-invariant direct methods
Least-squares matrix: full	Secondary atom site location: difference Fourier map
*R*[*F*^2^ > 2σ(*F*^2^)] = 0.039	Hydrogen site location: inferred from neighbouring sites
*wR*(*F*^2^) = 0.111	H-atom parameters constrained
*S* = 1.03	*w* = 1/[σ^2^(*F*_o_^2^) + (0.0535*P*)^2^ + 0.2624*P*] where *P* = (*F*_o_^2^ + 2*F*_c_^2^)/3
2533 reflections	(Δ/σ)_max_ < 0.001
190 parameters	Δρ_max_ = 0.16 e Å^−^^3^
0 restraints	Δρ_min_ = −0.15 e Å^−^^3^

### Special details

**Table:**

Geometry. Bond distances, angles *etc*. have been calculated using the rounded fractional coordinates. All su's are estimated from the variances of the (full) variance-covariance matrix. The cell e.s.d.'s are taken into account in the estimation of distances, angles and torsion angles
Refinement. Refinement of *F*^2^ against ALL reflections. The weighted *R*-factor *wR* and goodness of fit *S* are based on *F*^2^, conventional *R*-factors *R* are based on *F*, with *F* set to zero for negative *F*^2^. The threshold expression of *F*^2^ > σ(*F*^2^) is used only for calculating *R*-factors(gt) *etc*. and is not relevant to the choice of reflections for refinement. *R*-factors based on *F*^2^ are statistically about twice as large as those based on *F*, and *R*- factors based on ALL data will be even larger.

### Fractional atomic coordinates and isotropic or equivalent isotropic displacement parameters (Å^2^)

**Table:**

	*x*	*y*	*z*	*U*_iso_*/*U*_eq_	
O1	0.89839 (10)	0.06939 (6)	0.83483 (14)	0.0559 (4)	
O2	0.91991 (11)	0.19318 (7)	0.92712 (18)	0.0737 (5)	
N1	0.85664 (10)	0.16926 (7)	0.62191 (18)	0.0510 (4)	
C1	0.81409 (12)	−0.02577 (8)	0.3762 (2)	0.0436 (5)	
C2	0.75736 (14)	−0.01136 (10)	0.1926 (2)	0.0563 (5)	
C3	0.73596 (18)	−0.07145 (12)	0.0667 (3)	0.0715 (7)	
C4	0.77142 (19)	−0.14822 (12)	0.1148 (3)	0.0767 (8)	
C5	0.82829 (16)	−0.16422 (10)	0.2880 (3)	0.0656 (7)	
C6	0.85044 (12)	−0.10406 (8)	0.4248 (2)	0.0488 (5)	
C7	0.90596 (13)	−0.11995 (8)	0.6071 (2)	0.0523 (5)	
C8	0.92163 (13)	−0.06237 (8)	0.7386 (2)	0.0502 (5)	
C9	0.88322 (12)	0.01469 (8)	0.6891 (2)	0.0435 (4)	
C10	0.83343 (11)	0.03472 (8)	0.51467 (19)	0.0406 (4)	
C11	0.79834 (12)	0.11910 (8)	0.4725 (2)	0.0434 (4)	
C12	0.66655 (12)	0.13238 (8)	0.4466 (2)	0.0459 (5)	
C13	0.61359 (16)	0.18002 (12)	0.3073 (3)	0.0771 (7)	
C14	0.49394 (18)	0.19161 (15)	0.2832 (3)	0.0978 (9)	
C15	0.42673 (16)	0.15621 (12)	0.3962 (3)	0.0782 (7)	
C16	0.47770 (16)	0.10947 (12)	0.5371 (3)	0.0766 (7)	
C17	0.59754 (15)	0.09745 (10)	0.5628 (3)	0.0653 (6)	
C18	0.89155 (13)	0.14824 (9)	0.7974 (2)	0.0523 (5)	
H1	0.86962	0.21742	0.59296	0.0612*	
H2	0.73438	0.03977	0.15747	0.0675*	
H3	0.69733	−0.06099	−0.05240	0.0857*	
H4	0.75608	−0.18873	0.02799	0.0920*	
H5	0.85317	−0.21554	0.31735	0.0787*	
H7	0.93233	−0.17084	0.63780	0.0627*	
H8	0.95707	−0.07359	0.85922	0.0602*	
H11	0.82686	0.13419	0.35624	0.0520*	
H13	0.65851	0.20496	0.22768	0.0926*	
H14	0.45948	0.22429	0.18762	0.1174*	
H15	0.34624	0.16371	0.37798	0.0938*	
H16	0.43200	0.08537	0.61676	0.0919*	
H17	0.63155	0.06536	0.65983	0.0783*	

### Atomic displacement parameters (Å^2^)

**Table:**

	*U*^11^	*U*^22^	*U*^33^	*U*^12^	*U*^13^	*U*^23^
O1	0.0719 (7)	0.0430 (6)	0.0505 (6)	−0.0007 (5)	0.0006 (5)	−0.0011 (5)
O2	0.0814 (9)	0.0577 (7)	0.0769 (8)	0.0065 (6)	−0.0061 (7)	−0.0244 (6)
N1	0.0508 (7)	0.0327 (6)	0.0675 (9)	−0.0037 (5)	0.0017 (6)	0.0014 (6)
C1	0.0422 (8)	0.0404 (8)	0.0512 (8)	−0.0057 (6)	0.0170 (6)	−0.0019 (6)
C2	0.0645 (10)	0.0564 (9)	0.0502 (9)	−0.0054 (8)	0.0162 (7)	−0.0021 (8)
C3	0.0857 (13)	0.0788 (13)	0.0523 (10)	−0.0123 (10)	0.0180 (9)	−0.0131 (9)
C4	0.0951 (15)	0.0696 (12)	0.0709 (13)	−0.0161 (10)	0.0309 (11)	−0.0278 (10)
C5	0.0740 (12)	0.0438 (9)	0.0859 (13)	−0.0071 (8)	0.0352 (10)	−0.0134 (9)
C6	0.0459 (8)	0.0381 (8)	0.0669 (10)	−0.0049 (6)	0.0236 (7)	−0.0035 (7)
C7	0.0479 (8)	0.0346 (7)	0.0771 (11)	0.0021 (6)	0.0187 (8)	0.0068 (7)
C8	0.0462 (8)	0.0431 (8)	0.0610 (9)	0.0017 (6)	0.0067 (7)	0.0122 (7)
C9	0.0435 (8)	0.0375 (7)	0.0498 (8)	−0.0027 (6)	0.0080 (6)	0.0004 (6)
C10	0.0374 (7)	0.0352 (7)	0.0506 (8)	−0.0022 (5)	0.0113 (6)	0.0032 (6)
C11	0.0459 (8)	0.0354 (7)	0.0491 (8)	−0.0030 (6)	0.0077 (6)	0.0045 (6)
C12	0.0459 (8)	0.0357 (7)	0.0553 (9)	−0.0012 (6)	0.0042 (6)	0.0004 (6)
C13	0.0565 (10)	0.0874 (13)	0.0853 (13)	0.0041 (9)	0.0026 (9)	0.0366 (11)
C14	0.0629 (12)	0.1150 (18)	0.1095 (17)	0.0172 (12)	−0.0088 (12)	0.0423 (14)
C15	0.0478 (10)	0.0796 (13)	0.1042 (15)	0.0064 (9)	0.0009 (10)	−0.0069 (12)
C16	0.0562 (11)	0.0755 (12)	0.1031 (15)	−0.0015 (9)	0.0287 (10)	0.0056 (11)
C17	0.0546 (10)	0.0646 (11)	0.0795 (12)	0.0073 (8)	0.0192 (8)	0.0200 (9)
C18	0.0481 (8)	0.0421 (8)	0.0653 (10)	0.0026 (6)	0.0034 (7)	−0.0061 (8)

### Geometric parameters (Å, °)

**Table:**

O1—C9	1.3958 (17)	C12—C17	1.373 (2)
O1—C18	1.3617 (18)	C12—C13	1.366 (3)
O2—C18	1.2167 (19)	C13—C14	1.384 (3)
N1—C11	1.4622 (19)	C14—C15	1.346 (3)
N1—C18	1.3259 (19)	C15—C16	1.358 (3)
N1—H1	0.8600	C16—C17	1.387 (3)
C1—C2	1.417 (2)	C2—H2	0.9300
C1—C10	1.4280 (19)	C3—H3	0.9300
C1—C6	1.4191 (19)	C4—H4	0.9300
C2—C3	1.365 (3)	C5—H5	0.9300
C3—C4	1.392 (3)	C7—H7	0.9300
C4—C5	1.358 (3)	C8—H8	0.9300
C5—C6	1.418 (2)	C11—H11	0.9800
C6—C7	1.409 (2)	C13—H13	0.9300
C7—C8	1.356 (2)	C14—H14	0.9300
C8—C9	1.4075 (19)	C15—H15	0.9300
C9—C10	1.355 (2)	C16—H16	0.9300
C10—C11	1.5039 (19)	C17—H17	0.9300
C11—C12	1.525 (2)		
			
C9—O1—C18	120.13 (11)	C15—C16—C17	120.35 (19)
C11—N1—C18	126.78 (12)	C12—C17—C16	120.75 (18)
C11—N1—H1	117.00	O2—C18—N1	125.73 (14)
C18—N1—H1	117.00	O1—C18—O2	117.21 (13)
C2—C1—C6	118.33 (13)	O1—C18—N1	117.05 (13)
C6—C1—C10	118.94 (13)	C1—C2—H2	120.00
C2—C1—C10	122.72 (13)	C3—C2—H2	120.00
C1—C2—C3	120.94 (16)	C2—C3—H3	120.00
C2—C3—C4	120.63 (19)	C4—C3—H3	120.00
C3—C4—C5	120.27 (19)	C3—C4—H4	120.00
C4—C5—C6	121.17 (16)	C5—C4—H4	120.00
C5—C6—C7	122.02 (14)	C4—C5—H5	119.00
C1—C6—C5	118.64 (14)	C6—C5—H5	119.00
C1—C6—C7	119.33 (13)	C6—C7—H7	119.00
C6—C7—C8	121.20 (13)	C8—C7—H7	119.00
C7—C8—C9	118.75 (13)	C7—C8—H8	121.00
O1—C9—C10	122.00 (12)	C9—C8—H8	121.00
O1—C9—C8	114.88 (12)	N1—C11—H11	108.00
C8—C9—C10	123.11 (13)	C10—C11—H11	108.00
C1—C10—C11	121.82 (12)	C12—C11—H11	108.00
C1—C10—C9	118.59 (12)	C12—C13—H13	120.00
C9—C10—C11	119.58 (12)	C14—C13—H13	120.00
C10—C11—C12	113.63 (11)	C13—C14—H14	120.00
N1—C11—C12	110.68 (11)	C15—C14—H14	120.00
N1—C11—C10	108.56 (11)	C14—C15—H15	120.00
C11—C12—C13	120.86 (14)	C16—C15—H15	120.00
C11—C12—C17	121.24 (13)	C15—C16—H16	120.00
C13—C12—C17	117.89 (15)	C17—C16—H16	120.00
C12—C13—C14	120.81 (18)	C12—C17—H17	120.00
C13—C14—C15	120.9 (2)	C16—C17—H17	120.00
C14—C15—C16	119.30 (18)		
			
C18—O1—C9—C8	164.13 (13)	C5—C6—C7—C8	−176.94 (15)
C18—O1—C9—C10	−16.9 (2)	C6—C7—C8—C9	−1.2 (2)
C9—O1—C18—O2	−170.95 (14)	C7—C8—C9—O1	177.44 (13)
C9—O1—C18—N1	7.8 (2)	C7—C8—C9—C10	−1.5 (2)
C18—N1—C11—C10	−27.00 (19)	O1—C9—C10—C1	−175.98 (12)
C18—N1—C11—C12	98.36 (16)	O1—C9—C10—C11	3.0 (2)
C11—N1—C18—O1	15.8 (2)	C8—C9—C10—C1	2.9 (2)
C11—N1—C18—O2	−165.56 (15)	C8—C9—C10—C11	−178.08 (13)
C6—C1—C2—C3	1.4 (2)	C1—C10—C11—N1	−164.84 (12)
C10—C1—C2—C3	−177.10 (16)	C1—C10—C11—C12	71.56 (17)
C2—C1—C6—C5	−0.1 (2)	C9—C10—C11—N1	16.17 (17)
C2—C1—C6—C7	−179.36 (14)	C9—C10—C11—C12	−107.43 (15)
C10—C1—C6—C5	178.41 (14)	N1—C11—C12—C13	99.58 (17)
C10—C1—C6—C7	−0.9 (2)	N1—C11—C12—C17	−79.96 (17)
C2—C1—C10—C9	176.78 (14)	C10—C11—C12—C13	−137.98 (16)
C2—C1—C10—C11	−2.2 (2)	C10—C11—C12—C17	42.48 (19)
C6—C1—C10—C9	−1.7 (2)	C11—C12—C13—C14	179.58 (17)
C6—C1—C10—C11	179.35 (13)	C17—C12—C13—C14	−0.9 (3)
C1—C2—C3—C4	−1.2 (3)	C11—C12—C17—C16	−179.52 (16)
C2—C3—C4—C5	−0.2 (3)	C13—C12—C17—C16	0.9 (3)
C3—C4—C5—C6	1.5 (3)	C12—C13—C14—C15	−0.1 (3)
C4—C5—C6—C1	−1.3 (3)	C13—C14—C15—C16	0.9 (3)
C4—C5—C6—C7	177.92 (17)	C14—C15—C16—C17	−0.9 (3)
C1—C6—C7—C8	2.3 (2)	C15—C16—C17—C12	−0.1 (3)

### Hydrogen-bond geometry (Å, °)

**Table:**

Cg2 is the centroid of the C1–C6 phenyl ring.

**Table:**

*D*—H···*A*	*D*—H	H···*A*	*D*···*A*	*D*—H···*A*
N1—H1···O2^i^	0.86	2.07	2.8698 (17)	155
C8—H8···O1^ii^	0.93	2.58	3.4725 (18)	161
C16—H16···Cg2^iii^	0.93	2.92	3.722 (2)	145

Symmetry codes: (i) *x*, −*y*+1/2, *z*−1/2; (ii) −*x*+2, −*y*, −*z*+2; (iii) −*x*+1, −*y*, −*z*+1.
